# What time-lag for a retraction search on PubMed?

**DOI:** 10.1186/1756-0500-7-395

**Published:** 2014-06-25

**Authors:** Evelyne Decullier, Laure Huot, Hervé Maisonneuve

**Affiliations:** 1 Hospices Civils de Lyon, Pôle Information Médicale Evaluation Recherche, Unité de Recherche Clinique, Lyon F-69003, France; 2 Université de Lyon, EAM Santé Individu Société 4128, Lyon F-69003, France; 3 Université Lyon 1, Lyon F-69003, France; 4 Public Health Department, Paris-Sud 11 University, Paris, France

**Keywords:** Retraction, Time-lag

## Abstract

**Background:**

To investigate fraud and errors, scientists have studied cohorts of retraction notices. These researches have been performed using a PubMed search on publication type “retraction of publication” which retrieves the notices of the retractions. We assessed the stability of the indexation of retraction notices over a 15-month period and what was the time-lag to get stability.

**Findings:**

A search on notices of retraction issued in 2008 was repeated every 3 months during 15 months from February 2011. The first search resulted in 237 notices of retraction. Throughout the study period, 14 discrepancies with the initial search were observed (6%). We found that the number of retraction notices became stable 35 months after the retraction.

**Conclusions:**

The time-lag observed in this study has to be taken into account when performing a PubMed search.

## Findings

To investigate fraud and errors, scientists have studied cohorts of retraction notices [1-6]. These researches have been performed using a PubMed search on publication type “retraction of publication” which retrieves the notices of the retractions. The ability to find all retraction notices published in a given period is essential for these researches, and these studies rely on the fact that all retraction notices are identified as such in PubMed records. If the notification in the journal is labeled as a retraction or withdrawal, NLM will index it as a retraction (http://www.nlm.nih.gov/pubs/factsheets/errata.html). However, indexation process could also be prone to errors. Accuracy of indexation could not be assessed since it would require to have access to the full population of retraction notices, which is actually unknown. We therefore decided to assess the stability of the indexation of retraction notices over a 15-month period and what was the corresponding time-lag.

An initial search on the publication type “retraction of publication” issued in 2008 was performed [6] (“*retraction of publication*”[*Publication Type*] *AND* (“*2008*”[*PDAT*]: “*2008*”[*PDAT*])). We then repeated it every 3 months during 15 months, from February 2011. Each search was compared to the previous one to find discrepancies, which were classified as: newly identified retraction notice (not indexed in the former search) or change in the retraction’s authors. We tried to identify the reason for these changes. These classifications were not defined a priori.

The first search performed in February 2011 resulted in 237 notices of retraction published for the year 2008. Throughout the study period, 14 discrepancies with the initial search were observed (6%).

Firstly, 9 notices were newly identified, the last appearing 9 months after the first search (Table 1). Among these, the word “retraction” was present in the title for 6 at the time of the search. Concerning the explanation for the late indexation, in 7 cases, although the e-publication date was in 2008, the publication date of the retraction notice was in 2011, certainly leading to the update of the PubMed record with re-indexation (see Table 2 for an example). In one case, a correction to the retraction notice was issued in 2011, certainly leading to the modification of the initial indexation of the notice. For 1 case, we could not find any explanation for the late indexation.

**Table 1:** Quaterly PubMed searches during 15 months on 2008 retraction notices

**Download date**	#** citations**	**Citations modified compared to previous search**			**Coding**
feb-2011	237				
22-may 2011	239	Wolfort, R.M., Manriquez, R., Stokes, K.Y., Granger, D.N.	Retraction : Platelet-derived RANTES mediates hypercholesterolemia-induced superoxide production and endothelial dysfunction	Arterioscler Thromb Vasc Biol	**Newly identified**
		Wolfort,R.M., Manriquez, R., Stokes, K.Y., Granger, D.N.	Platelet-derived RANTES mediates hypercholesterolemia-induced superoxide production and endothelial dysfunction: retraction	Arterioscler Thromb Vasc Biol	
22-aug-2011	241		Retraction	J Am Soc Nephrol	**Newly identified**
			Retraction notice to “Quantitative role of p42/44 and p38 in the production and regulation of cytokines TNF-alpha, IL-1beta and IL-12 by murine peritoneal macrophages in vitro by Concanavalin A “[Cytokine 2007;37:62–70]”	Cytokine	
			Retraction : Platelet-derived RANTES mediates hypercholesterolemia-induced superoxide production and endothelial dysfunction	Arterioscler Thromb Vasc Biol	**Authors**
22-nov-11	246	Toggweiler, S., Erne, P.	Functional mitral stenosis--a rare complication of the Impella assist device	Eur J Echocardiogr	**Newly identified**
		Namboodri, N.	Doppler echocardiographic assessment of TTK Chitra prosthetic heart valve in the mitral position	Eur J Echocardiogr	
		Reiner, J. L., Nakayama, S. F., Delinsky, A. D., Strynar, M. J., Lindstrom, A. B.	Retraction. Method development and measurement of perfluorinated compounds in U.S. chicken eggs	Environ Sci Technol	
		Oka, H., Yoshioka, M., Morita, M., Onouchi, K., Mochio, S., Inoue, K.	Retractions: “Cardiovascular dysautonomia in de nove Parkinson’s disease” J Neurol Sci 2006; 241:59–65 and “Cardiovascular autonomic dysfunction in dementia with Lewy bodies and Parkinson’s disease” J Neurol Sci 2007; 254:72–77.	J Neurol Sci	
		Ho, S.	Structure and anatomy of the aortic root	Eur J Echocardiogr	
22-feb-2012	246		Doppler echocardiographic assessment of TTK Chitra prosthetic heart valve in the mitral position	Eur J Echocardiogr	**Authors**
			Functional mitral stenosis--a rare complication of the Impella assist device	Eur J Echocardiogr	
			Structure and anatomy of the aortic root	Eur J Echocardiogr	
23-may-2012	246		Retraction. Method development and measurement of perfluorinated compounds in U.S. chicken eggs	Environ Sci Technol	**Authors**

**Table 2:** **Example of an e**-publication date in 2008 and a publication date in 2011 and the corresponding Medline indexation*

**Reference: Environ Sci Technol. 2011 Sep 15; 45 (18):7949. Epub 2008 Jul 23.**	
PMID	21910498
OWN	NLM
STAT	MEDLINE
DA	20110913
DCOM	20120308
IS	1520-5851 (Electronic)
IS	0013-936X (Linking)
VI	45
IP	18
DP	**2011 Sep 15 (publication date)**
TI	Retraction. Method development and measurement of perfluorinated compounds in U.S. chicken eggs
PG	7949
LA	eng
PT	Retraction of Publication
DEP	**20080723 (e-publication date)**
PL	United States
TA	Environ Sci Technol
JT	Environmental science & technology
JID	0213155
SB	IM
ROF	Environ Sci Technol. doi:10.1021/es800770f
EDAT	**2011/09/14 06:00 (Input date, or publication date when recorded more than 12 months after publication)**
MHDA	2012/03/09 06:00
CRDT	2011/09/14 06:00
PHST	2008/07/23 [aheadofprint]
AID	10.1021/es800770f [doi]
PST	ppublish
SO	Environ Sci Technol. 2011 Sep 15;45(18):7949. Epub 2008 Jul 23.

*PubMed link explaining fields: http://www.nlm.nih.gov/bsd/mms/medlineelements.html#edat.

Secondly, a total of 5 discrepancies on the author list was observed. They consisted in the deletion of the author list initially available (Table 1). All these modifications occurred in notices which were newly identified during our study.

We found that the number of retraction notices became stable in November 2011 for the retraction notices of the year 2008 i.e., 35 months after. This result shows that retraction notices, despite being a very specific entity, are not always indexed as “retraction of publication” in PubMed. However, as raised by Ivan Oransky (http://retractionwatch.com/), there is no other available database for retractions.

The time-lag observed in this study has to be taken into account when performing a PubMed search and a time-lag of at least 3 years should be respected between the time of the search and the year of interest.

Errors in indexation were corrected when the PubMed record had to be updated (publication, erratum), consequently we cannot ascertain that all retraction notices are indexed as such. Therefore, to ease indexation process, retraction notices titles should at least include the word “retraction” as recommended by the Committee on Publication Ethics (COPE) [7]. Furthermore, the use of a standard retraction form would be very useful as it could help to standardize the title as well as the way of presenting authors for retractions [6].

## Competing interest

The authors declare that they have no competing interests.

## Authors’ contributions

ED declares that she designed the study, performed the searches, analysed the data, interpreted the data and wrote the manuscript. LH declares that she designed the study, participated in drafting the manuscript and that she has read and approved the final version. HM declares that he participated in drafting the manuscript and that he has read and approved the final version. All authors read and approved the final manuscript.

## Authors’ information

ED is a senior researcher (PhD), LH is a senior researcher (PharmD, PhD), HM is a senior researcher (MD).
